# The Clinical and Economic Impact of Biologic Agents in Asthma Management: a Systematic Review

**DOI:** 10.1007/s00408-026-00903-0

**Published:** 2026-06-16

**Authors:** Cataldo Procacci, Maria Rosaria Gualano, Leonarda Maurmo, Ilaria Valentini, Sofia Mao, Walter Ricciardi

**Affiliations:** 1 Pharmaceutical Department, Local Health Autority of BAT, Andria, Italy; 2 Graduate School of Health Economics and Management (ALTEMS), Università Cattolica del Sacro Cuore, Rome, Italy; 3 UniCamillus–Saint Camillus International University of Health and Medical Sciences, Rome, Italy; 4 Università di Foggia, Foggia, Italy; 5 Section of Hygiene, University Department of Life Sciences and Public Health, Università Cattolica del Sacro Cuore, Rome, Italy

## Abstract

**Objectives:**

Biologic therapies represent transformative interventions for severe asthma; however, comprehensive integration of clinical effectiveness with economic evidence across real-world populations remains incomplete. This systematic review synthesizes both domains to support clinical and policy decisions.

**Methods:**

Systematic literature search (PubMed, Scopus; 2014–2024) following PRISMA 2020 guidelines with prospective PROSPERO registration (CRD420251153385). Eligible studies evaluated biologic therapies in real-world asthma populations, reporting clinical outcomes (exacerbations, lung function, asthma control, quality of life) and economic measures (costs, ICER, healthcare resource utilization). GRADE methodology assessed evidence certainty; risk of bias evaluated using RoB 2 (RCTs) and ROBINS-I (observational studies).

**Results:**

Twenty-seven studies (25 observational, 2 RCTs; 59,958 patients) evaluated omalizumab (*n* = 11), mepolizumab (*n* = 5), benralizumab (*n* = 4), dupilumab (*n* = 1), tezepelumab (*n* = 1), reslizumab (*n* = 1) and multiple biologic drugs comparatively (*n* = 4). All agents showed exacerbation reductions (46–86%), with 48–81% of previously exacerbating patients achieving exacerbation-free status. Hospitalizations decreased 57–85% and emergency department visits reduced 52–72%. Oral corticosteroid-dependent patients decreased 53–67%, representing substantial safety and quality-of-life benefits. Healthcare resource utilization reductions generated cost offsets of €1,181–2469 per patient annually, achieving favorable cost-effectiveness (€602–2244 per exacerbation avoided). High treatment persistence (51–54 months) and adherence (70–94.6%) exceeded conventional therapies. Significant methodological limitations were evident: 80.8% observational studies had serious bias risk.

**Conclusions:**

Biologic therapies achieve substantial clinical benefits and favorable economic value through healthcare cost offsets. Precision medicine approaches and early response assessment optimize patient selection and clinical outcomes in severe asthma management.

## Introduction

 Asthma represents one of the most prevalent chronic respiratory diseases globally, affecting over 300 million individuals worldwide and constituting a significant public health burden [1]. The economic implications are substantial, with healthcare systems facing mounting pressure from both direct medical costs and indirect societal costs associated with this condition. In the United States alone, asthma imposes an estimated annual economic burden of $82 billion, with projections suggesting that uncontrolled asthma will cost nearly $964 billion over the next two decades [2, 3]. Similarly, healthcare systems in both Europe and Asia bear considerable costs, with estimates ranging from approximately € 980 (1734 CNY) per patient annually in China to over €5600 in severe European cases requiring regular oral corticosteroid treatment [4, 5]. 

Asthma exhibits heterogeneous phenotypes with variable symptom presentation and airflow limitation across patients, necessitating complex management approaches. Personalized, patient-centered treatment strategies tailored to individual characteristics are essential for optimizing clinical outcomes and quality of life [6]. Contemporary asthma management paradigms emphasize the importance of achieving optimal disease control through comprehensive care models that integrate clinical effectiveness with economic efficiency [7]. However, despite the availability of evidence-based treatment guidelines and effective therapeutic options, a substantial proportion of patients with asthma fail to achieve adequate disease control, resulting in preventable exacerbations, increased healthcare resource utilization, and diminished quality of life [8]. 

Economic evaluation of asthma management strategies is critical for optimizing resource allocation, employing methodological approaches including cost-effectiveness, cost-utility, and cost-benefit analyses that assess outcomes using standardized measures such as quality-adjusted life years (QALYs) [9, 10]. Furthermore, many economic models fail to adequately capture disease heterogeneity, phenotypic variations, and real-world clinical practice patterns, limiting their applicability to precision medicine approaches [11, 12]. 

Healthcare resource utilization patterns in asthma demonstrate clear associations with disease severity and control status, with severe uncontrolled asthma patients exhibiting disproportionately higher healthcare costs and resource consumption [2]. Direct medical costs encompass hospitalizations, emergency department visits, outpatient consultations, diagnostic procedures, and pharmacological treatments, while indirect costs include productivity losses, work absenteeism, and caregiver burden. Mean costs of asthma-related medical services and medications increased in regard to disease severity [13]. The relationship between asthma control and economic outcomes is well-established, with well-controlled asthma associated with significantly lower healthcare resource utilization and reduced direct and indirect costs compared to poorly controlled disease [3]. 

Biological therapies for severe asthma represent a particular area of economic interest, given their substantial acquisition costs and demonstrated clinical benefits in carefully selected patient populations [14]. Cost-effectiveness analyses of biological agents reveal varying economic profiles across different healthcare systems and patient populations [15]. While these therapies consistently demonstrate clinical efficacy in reducing exacerbations and improving quality of life, their cost-effectiveness ratios often exceed conventional willingness-to-pay thresholds, particularly in middle-income countries with limited healthcare budgets [16]. 

The pediatric asthma population presents unique economic considerations, the increase of disease severity is associated with exponentially higher healthcare costs and resource utilization [17]. Cost ratios for children with severe asthma can exceed 3.4 times those of matched controls, with hospitalization representing the primary cost driver [18]. Furthermore, pediatric asthma imposes significant indirect costs through parental work productivity losses and educational disruption, highlighting the broader societal impact of inadequate disease management [19].

Evaluating asthma management is crucial given high costs and evolving treatments. These insights are essential for guiding policy and clinical practice. This literature review aims to systematically evaluate and compare the clinical and economic outcomes of biologic treatments, with particular emphasis on healthcare resource utilization patterns and cost-effectiveness profiles. This review seeks to provide healthcare decision-makers with comprehensive evidence to guide the implementation of cost-effective asthma care delivery models that optimize both patient outcomes and resource allocation.

## Methods

### Study Design and Protocol Registration

This systematic review was designed and conducted in accordance with the PRISMA 2020 (Preferred Reporting Items for Systematic Reviews and Meta-Analyses) guidelines and the Cochrane Handbook for Systematic Reviews of Interventions [20]. The review protocol was prospectively registered on PROSPERO (International Prospective Register of Systematic Reviews) with registration number CRD420251153385 before initiating the search and study selection process.

The review question was developed using the Population, Intervention, Comparison, Outcome, type of study (PICOS) framework [21]: among patients with asthma (P) in real-world settings, how do all biologic therapies perform in terms of clinical outcomes and economic outcomes (O)?

### Eligibility Criteria for Study Selection

#### Population

Studies conducted on patients with a clinical diagnosis of asthma, regardless of disease severity, were included. Studies conducted in real-world clinical practice settings were considered, including primary, secondary, and tertiary care contexts (outpatient clinics, general medicine clinics, specialized hospital clinics in pulmonology and allergy/immunology, emergency departments, and inpatient wards). Studies conducted in both publicly funded healthcare systems (e.g., National Health Services) and mixed public-private insurance models were included, utilizing national health databases, electronic health records, insurance claims data, and patient registries from real-world practice environments.

#### Interventions

All biologic therapies for the management of asthma were considered, both for single conditions and multiple conditions. Studies evaluating patients receiving the same drug for asthma and other indications were included.

#### Comparison

Eligible studies were required to include a comparative assessment of biologic therapies conducted in real-world clinical settings. Studies that do not offer comparative data or alternative management strategies were excluded.

#### Outcomes

The outcomes considered were classified into the following categories:

- Clinical outcomes: asthma control, frequency of exacerbations, symptom reduction, improvement in pulmonary function (forced expiratory volume in one second [FEV₁], FEV₁/forced vital capacity [FVC], peak expiratory flow [PEF]), reduction of nocturnal symptoms, therapy adherence (assessed using quantitative measures such as medication possession ratio, proportion of days covered, or composite adherence scores), quality of life (measured with validated asthma-specific or generic questionnaires).
- Economic outcomes: direct healthcare costs (hospitalizations, outpatient visits, medications), indirect costs (productivity loss, work and school absenteeism), incremental cost-effectiveness ratios (ICER), standardized mean differences for continuous variables, access to healthcare services, reduction in overall treatment costs.

#### Study Types

Primary research studies using real-world data from clinical practice were included, encompassing both randomized controlled trials (RCTs) and non-randomized studies. Specifically:

- Retrospective cohort studies analyzing real-world patient outcomes and healthcare resource utilization.
- Cross-sectional studies examining economic burden and resource consumption patterns.
- Cost-effectiveness analyses (CEA), cost-utility analyses (CUA), cost-benefit analyses (CBA), and cost-consequence analyses (CCA) based on real-world data.

### Excluded Criteria

The exclusion criteria established for the review were as follows: case reports, case series, letters to the editor, editorials, narrative reviews not providing quantitative data, economic models based exclusively on data derived from registrational clinical trials without a real-world component.

### Data Sources and Search Strategy

A systematic literature search was conducted in the following electronic databases: PubMed/MEDLINE (National Library of Medicine) and Scopus (Elsevier). The search covered the time period from 01 January 2014 to 31 December 2024, with the aim of identifying recent evidence in the field of pharmacological management of asthma with clinical and economic outcomes.

The search strategy was developed using a combination of MeSH (Medical Subject Headings) terms, free text terms, and Boolean operators. The complete strategy for each database was uploaded and made available in the PROSPERO registry. No restrictions related to regulatory or experimental environments were applied. In addition to searching electronic databases, reference lists of included studies were examined to identify additional relevant studies not identified by the primary search (backward citation searching). Experts in the field were also contacted to identify any unpublished studies or relevant grey literature.

### Study Selection

The study selection process was conducted independently by two reviewers following a standardized two-phase approach. Two reviewers independently examined the titles and abstracts of all records identified through the literature search, applying predefined eligibility criteria. Potentially relevant studies were selected for full-text review. The full texts of potentially relevant studies were retrieved and independently assessed by four reviewers to confirm eligibility according to predetermined criteria. Any discrepancies in data extraction between reviewers were discussed and resolved through consensus or consultation with a third reviewer.

The study selection process was documented using the PRISMA 2020 flow diagram, reporting the number of records identified, excluded, and included at each stage, with corresponding reasons for exclusion.

### Data Extraction and Synthesis

Data extraction from included studies was performed independently by at least two reviewers using a standardized and pre-tested data extraction form. The form was piloted on a sample of studies to verify the completeness and clarity of the items to be extracted.

From each study, were extracted details on study design/setting and objectives, participant characteristics, interventions and comparators, outcomes (clinical and economic) with their definitions and measurement methods, and quantitative results for effectiveness and costs (including resource use and cost-effectiveness metrics).

A systematic, structured, and transparent narrative synthesis was used to summarize and interpret the results of included studies, maintaining methodological rigor. The narrative synthesis followed a thematic approach, organizing results by type of pharmacological intervention, evaluated outcome, and healthcare setting. The characteristics of included studies, studied populations, evaluated interventions, and main clinical and economic results were described.

### Risk of Bias Assessment and Certainty of Evidence Evaluation

The certainty of evidence was systematically classified into four levels (high, moderate, low, very low) using GRADE methodology [22], applied differentially by study design. For randomized controlled trials (RCTs), the initial certainty level was high with potential downgradng across five RoB 2 domaine [23]. For observational studies, the initial level was low, allowing downgrading or upgrading when robust effect indicators were present.

### Ethical Considerations

Since this systematic review is based exclusively on data already published and available in the literature, it was not necessary to obtain approval from an ethics committee. All studies included in the review obtained the necessary ethical approvals at the time of their original conduct, as reported in their respective publications.

## Results

### Study Selection and Characteristics

**Fig. 1:**
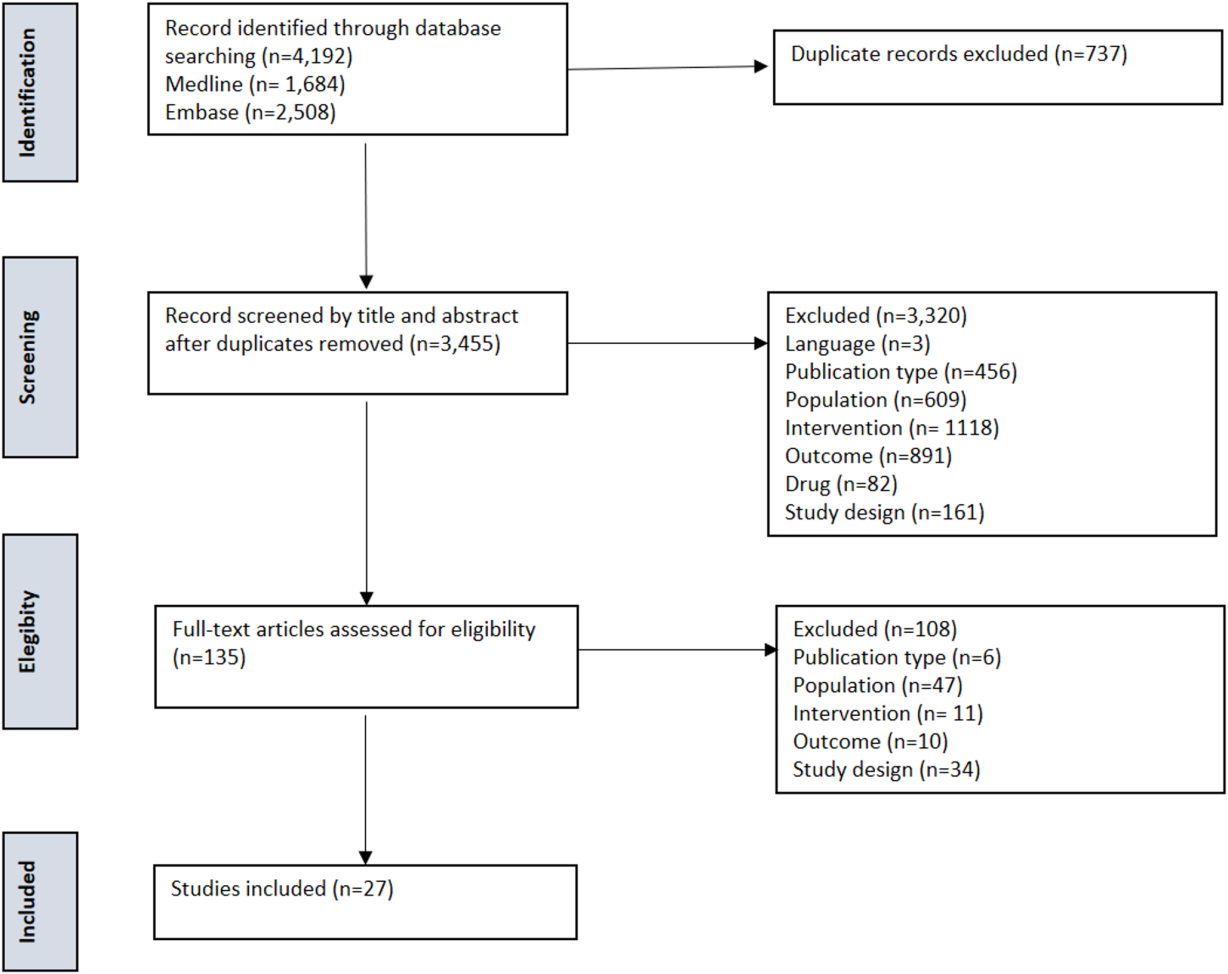
Prisma flow chart

The initial search yielded 4192 records across the targeted databases. After removing 1481 duplicate records, 3455 were kept for screening. From these articles, 3,330 were excluded after reading title and/or abstract. Thus, 135 studies were obtained, from which, after reading the full text, 108 were excluded for several reasons. See the PRISMA flow diagram (Fig. 1) for more details.

Of the 27 eligible studies, 25 (92.6%) were observational real-world studies, while only 2 (7.4%) were randomized controlled trials.

The included studies are conducted between 2014 and 2024. Geographic distribution revealed substantial representation from the United States (*n* = 4), Spain (*n* = 3), United Kingdom (*n* = 2), and Italy (*n* = 2), with additional studies conducted across multiple countries including Canada, Portugal, Taiwan, France, Poland, Turkey, Jordan, and Malta. Three studies included multiple nations.

Regarding the sample size, substantial differences were observed, with studies including from 12 to 32,748 participants. The studies encompassed a total of 59,958 patients, with a mean sample size of 2220 patients per study. Table 3

The majority of studies (*n* = 23, 85.2%) utilized a pre-post self-controlled design without a control group, comparing outcomes within the same patient cohort at different time points (before and after intervention). A smaller proportion of studies (*n* = 4, 14.8%) incorporated a control group comparison, allowing for between-group patient comparisons and enhanced causal inference through comparison across different patient populations.

### Characteristics of Pharmacological Interventions

The biologic therapies evaluated across the included studies demonstrated the following distribution: omalizumab was the most frequently studied agent (*n* = 11, 40.7%), followed by mepolizumab (*n* = 5, 18.5%), benralizumab (*n* = 4, 14.8%) and single studies each for dupilumab, tezepelumab and reslizumab. Four studies evaluated multiple biologic agents comparatively.

The predominant time horizon was 12 months pre- and post-intervention (*n* = 20 studies), enabling standardized comparison of clinical and economic outcomes. Extended follow-up periods revealed sustained benefits: Jackson et al. [24] reported benralizumab outcomes at 48 months, and Humbert et al. [25] analyzed persistence patterns over 24 months with observations extending to 3 years post-discontinuation Table 4.

Variable follow-up periods were employed in several studies to capture real-world treatment patterns. Bernstein et al. utilized both 12-month follow-up and variable follow-up (mean 1024 days, approximately 34 months) to assess short-term and long-term outcomes. Arrobas et al. employed an average 22-month follow-up (range 3.4–37 months) with retrospective 12-month pre-treatment assessment and prospective follow-up extending to 24 months.

### Results of the Risk-of-Bias Assessment

The risk-of-bias analysis of the included studies was conducted considering various methodological domains, as illustrated in Tables 1 and 2. The assessment allowing the identification of strengths and limitations in the design and the conduct of the analyzed clinical trials.

**Table 1:** Risk of bias assessment across five methodological domains using the RoB 2 tool for randomized controlled trials (*n* = 2)

Dominio	Low risk	Some concerns	High risk
Randomization process	2 studies	0	0
Deviations from interventions	0	2 studies	0
Missing outcome data	2 studies	0	0
Outcome measurement	2 studies	0	0
Selection of reported result	0	2 studies	0
Overall RoB	0	2 (100%)	0

**Table 2:** Risk of bias assessment across seven methodological domains using the ROBINS-I tool for observational studies (*n* = 25)

Domain	Low risk	Moderate risk	Serious risk	Critical risk
1. Confounding	0 (0%)	7 (28.0%)	18 (72.0%)	0 (0%)
2. Selection of participants	1 (4.0%)	9 (36.0%)	15 (60.0%)	0 (0%)
3. Classification of interventions	25 (100%)	0 (0%)	0 (0%)	0 (0%)
4. Deviations from interventions	3 (12.0%)	21 (84.0%)	1 (4.0%)	0 (0%)
5. Missing data	8 (32.0%)	16 (64.0%)	1 (4.0%)	0 (0%)
6. Measurement of outcomes	21 (84.0%)	4 (16.0%)	0 (0%)	0 (0%)
7. Selection of reported results	4 (16.0%)	21 (84.0%)	0 (0%)	0 (0%)
Overall RoB	0 (0%)	6 (24.0%)	19 (76.0%)	0 (0%)

**Table 3:** Main characteristic of the selected studies

Authors	Year	Country	RCT (Y/*N*)	Study design	Inclusion criteria	Drugs	Comparison	Sample size	Population age
Al-Lehebi RO, Al Ahmad M, Maturu VN, et al.	2024	Multiple (9 countries)	N	Multi-country, bi-directional, self-controlled, observational cohort study	Patients aged ≥ 18 years, clinical diagnosis of severe asthma, prescribed ≥ 1 dose of mepolizumab, 12 months of historical data prior to index date	Mepolizumab	Pre vs. post (self-controlled)	525	Mean age 49.6 (SD 13.4) years
Arrobas A, Barbosa MP, Rabiais S, et al.	2021	Portugal	N	Pragmatic cost-effectiveness analysis based on eXpeRience registry data	Patients with uncontrolled persistent allergic asthma from eXpeRience registry (*n* = 62)	Omalizumab	Pre vs. post (self-controlled)	62	Mean age 49.2 years
Bagnasco D, Povero M, Pradelli L, et al.	2021	Italy	N	Prospective and retrospective real-life study	Patients ≥ 18 years with severe uncontrolled eosinophilic asthma starting mepolizumab	Mepolizumab	Pre vs. post (self-controlled)	106	Mean age 57.35 years
Bernstein JA, Silver J, Packnett E, et al.	2024	USA	N	Retrospective cohort analysis using MarketScan Research Databases	Adult patients with CRSwNP initiating mepolizumab between November 2014 - September 2021, ≥ 12 months enrollment pre- and post-index, ≥ 2 claims for mepolizumab in first 6 months	Mepolizumab	Pre vs. post (self-controlled)	189	Mean age 51.1 (SD 11.4) years
Bhutani M, Yang WH, Hébert J et al.	2017	Canada	N	One-year open-label observational study	Moderate-to-severe allergic asthma, inadequately controlled despite high-dose ICS	Omalizumab	Pre vs. post (self-controlled)	99	Mean age 48 years
Carpagnano GE, Resta E, Povero M, et al.	2021	Italy	N	Retrospective multi-center observational study	Adult patients with uncontrolled severe eosinophilic asthma switching from omalizumab to mepolizumab	Omalizumab and mepolizumab	Pre vs. post (self-controlled)	33	Mean age 57 years
Casale TB, Luskin AT, Busse W, et al.	2019	USA	N	Prospective real-world study	Patients ≥ 12 years with allergic asthma candidates for omalizumab	Omalizumab	Pre vs. post (self-controlled)	801	Median age 49 years (12–100)
Casale T, Molfino NA, Silver J, et al.	2021	USA	N	Retrospective cohort study	Patients with severe asthma and ≥ 1 of 7 specific comorbidities initiating mepolizumab	Mepolizumab	Pre vs. post (self-controlled)	639	Mean age ~ 50 years across subgroups
Chapman KR, Cogger K, Arthurs E, et al.	2024	Canada	N	Retrospective, observational study using ICES database and patient support program	Patients initiating mepolizumab February 2016-March 2019, ≥ 18 years, 12 months continuous enrollment pre- and post-index	Mepolizumab	Pre vs. post (self-controlled)	275	Mean 57.6 (SD 13.5) years
Chen HC, Huang CD, Chang E, et al.	2016	Taiwan	N	Retrospective population-based database cohort study	Patients with moderate to severe asthma receiving omalizumab for > 4 months	Omalizumab	Pre vs. post (self-controlled)	282	Mean age 51.3 ± 17.2 years
Chen W, Tran TN, Sadatsafavi M, et al.	2023	Multiple (19 countries)	N	Propensity score-matched prospective cohort study	Adults ≥ 18 with severe asthma and high oral corticosteroid exposure	Various Biologics drugs (anti-IgE anti-IL-5/5R anti-IL-4Ra)	Pre vs. post (self-controlled)	996	Adults ≥ 18 years
Chung Y, Katial R, Mu F et al.	2022	USA	N	Retrospective cohort study using insurance claims database	Patients with asthma aged ≥ 12 years with ≥ 2 records of benralizumab and ≥ 2 exacerbation episodes pre-index	Benralizumab	Pre vs. post (self-controlled)	204	≥ 12 years, mean age 45.3 ± 16.6 years
Entrenas Costa LM, Casas-Maldonado F, Soto Campos JG, et al.	2019	Spain	N	Multicentre retrospective study	Patients aged ≥ 18 years with severe persistent allergic asthma, started omalizumab before 1 January 2014	Omalizumab	Pre vs. post (self-controlled)	220	≥18 years, Mean age 49.1 ± 13.8
Gouder C, West LM, Montefort S	2015	Malta	N	Open, prospective observational real-life study	Adult patients eligible for omalizumab for asthma	Omalizumab	Pre vs. post (self-controlled)	22	Mean age 52.7 ± 11 years
Humbert M, Bourdin A, Taillé C, et al.	2022	France	N	Nationwide drug utilization study using health administrative database	Asthma patients ≥ 6 years who initiated omalizumab for ≥ 16 weeks from 2009–2019	Omalizumab	Pre vs. post (self-controlled)	19,203	≥ 6 years, adults mean 52.1 ± 15.6 years, children 11.9 ± 3.2 years
Jahnz-Różyk K, Lis J, Warchoł M, Kucharczyk A et al.	2018	Poland	N	Retrospective observational registry study	Adults and adolescents with severe allergic asthma, uncontrolled despite high-dose ICS/LABA and/or other controllers, meeting Polish Drug Programme criteria for omalizumab	Omalizumab	Pre vs. post (self-controlled)	85	Mean age 44.9 years
Jackson DJ, Burhan H, Menzies-Gow A, et al.	2022	United Kingdom	N	Retrospective cohort study (real-world study)	Adults (≥ 18 years) with SEA inadequately controlled despite high-dosage ICS plus LABA, blood eosinophil ≥ 300 cells/mL, ≥ 3 asthma exacerbations requiring systemic corticosteroids in previous 12 months or treatment with continuous OCS over previous 6 months	Benralizumab	Pre vs. post (self-controlled) divided in predefined subgroups based on patients’ baseline characteristics	208	Mean age 50.5 years
Menzies-Gow A, Bourdin A, Chupp G et al.	2021	Multiple (18 countries)	Y	Randomized, double-blind, placebo-controlled trial	Adults (≥ 18 yrs) with severe eosinophilic asthma (≥ 150 eosinophils/µL at screening or ≥ 300 eosinophils/µL in previous year), ongoing high-dose ICS + additional controller	Tezepelumab	Placebo	1059 (528 tezempemumab and 531 placebo)	Mean: 50 years (range specified in manuscript)
Mosnaim G, Huang H, Ariely R, et al.	2020	USA	N	Retrospective claims database analysis	Patients aged ≥ 5 years with persistent asthma per HEDIS criteria, continuously enrolled for 24 months	Various Biologics drugs (anti-IgE anti-IL-5/5R anti-IL-4Ra)	2 mutually exclusive cohorts	32,748	≥ 5 years (mean: 41.5 ± 18.7)
Niven RM, Saralaya D, Chaudhuri R, et al.	2016	United Kingdom	N	Non-interventional, mixed methodology (retrospective and prospective data collection)	Adult patients (≥ 16 years) with severe persistent allergic asthma prescribed omalizumab	Omalizumab	Pre vs. post (self-controlled)	258	≥ 16 years (mean: 44.7 ± 14.2)
Padilla-Galo A, Moya Carmona I, Ausín P, et al.	2023	Spain	N	Multicenter, observational, retrospective cohort study	Adult patients (≥ 18 years) with severe eosinophilic asthma treated with benralizumab	Benralizumab	Pre vs. post (self-controlled)	204	≥ 18 years (mean: 56.4 ± 12.4)
Padilla-Galo A, García-Ruiz AJ, Levy Abitbol RC, et al.	2021	Spain	N	Cross-sectional multicentre study	Patients with refractory eosinophilic asthma who received treatment with benralizumab for at least 12 months	Benralizumab	Pre vs. post (self-controlled)	44	Mean age 56.4 ± 12.4 years
Tabaza, H, Abu Farha, R, Naser, AY et al.	2023	Jordan	N	Retrospective observational cohort study	Adults (age ≥ 18 years) diagnosed with asthma at least 1 year before hospital admission	Various Biologics drugs (anti-IgE anti-IL-5/5R anti-IL-4Ra)	Pre vs. post (self-controlled)	494	Median age 50.0 years (38.0–63.0 IQR)
Tugay D., Top M.t, Aydin Ö., et al.	2023	Turkey	N	Real-world patient-level cost-effectiveness analysis	Patients with severe allergic asthma treated at four major medical centers	Omalizumab	control group	206 patients (126 OML + SoC group, 80 SoC group)	19 years and older (median age 50.3 vs. 48.3 years)
Wechsler ME, Peters SP, Hill TD, et al.	2021	USA	N	Retrospective study using center- and panel-based medical chart review	Adults ≥ 18 years with severe eosinophilic asthma, treated with IV reslizumab ≥ 7 months, available medical records ≥ 6 months pre- to ≥ 7 months post-reslizumab initiation	Reslizumab	Pre vs. post (self-controlled)	215	Mean age 45.2 years (SD 11.9)
Wenzel S, Castro M, Corren J, et al.	2016	Multiple (16 countries)	Y	Randomised, double-blind, placebo-controlled, parallel-group, pivotal phase 2b dose-ranging trial	Adults (≥ 18 years) with asthma diagnosis ≥ 12 months, medium-to-high-dose inhaled corticosteroids plus long-acting β2 agonist, pre-bronchodilator FEV1 40–80% predicted, ACQ-5 score ≥ 1.5, reversibility ≥ 12% and 200 mL in FEV1 after salbutamol, history of systemic corticosteroid burst or hospitalization/emergency care for asthma in past year	Dupilumab	Placebo	769 patients (158 placebo group and 611 dupilumab)	Mean age 48.6 years (SD 13.0)
Yoshikawa H, Iwata M, Matsuzaki H, et al.	2016	Japan	N	retrospective, pre-post observational study	Pediatric patients with severe asthma who received omalizumab for 2 years	Omalizumab	Pre vs. post (self-controlled)	12	Mean age 12 years (range 9–14 years)

**Table 4:** Studies outcomes and perspective

Authors (year)	Clinical outcome	Economic (or organizative) outcomes	Results	Perspective	Time horizon
Al-Lehebi et al. (2024)	Reduction in clinically significant exacerbations, reduction of use of oral corticosteroid, improved lung function, improved asthma control	Reduction hospitalizations, reduction ER visits, decrease healthcare resource utilization.	76% CSE reduction, 36.1% oral corticosteroid-free status, and 64.5% well-controlled asthma—with favorable economic impact through reduced hospitalizations (22.5% to 6.9%) and ED visits, partially offset by increased physician monitoring visits.	Healthcare system	12 months pre-initiation and 12 months post-initiation of mepolizumab (mandatory 12-month follow-up after index date).
Arrobas et al. (2021)	Reduction in exacerbations, reduction in GETE score	ICER, total costs comparison pre vs. post-omalizumab	Clinically, 82.1% exacerbation reduction (8.to 1.5/patient-year) and 54.1% GETE score improvement. Economically, favorable ICERs (€2,244/exacerbation avoided, €1,750/GETE unit) offset by substantial cost increase (€3,023to €16,111/patient-year)	societal (direct and indirect costs)	Average 22 months (range 3.4–37 months); retrospective 12 months pre-treatment, prospective up to 24 months post-treatment
Bagnasco et al. (2021)	Reduction in exacerbations, reduction in OCS-dependent patients, rate of hospitalization	Total costs: exacerbation reduction, MEP annual cost, Working days lost, Patients losing working days.	81% exacerbation reduction (4.085 to 0.774/PY; 62% of savings). 61% reduction in OCS-dependent patients (79.2% to 33%; 4.73 mg daily dose reduction). Economically, 85.4% hospitalization rate reduction (0.38 to 0.057/PY; RR 0.146). 11.87 fewer work days lost annually (15 to 4 days); 51.2% reduction in affected patients (63.2 to 31.2%; 33% of savings). Total savings €2,469/patient (excluding MEP cost; funds 22% of annual cost).	societal (includes productivity)	12 months pre-treatment vs. 12 months post-treatment
Bernstein et al. (2024)	Reduction in NP-related exacerbations, reduction in asthma-related exacerbations, reduction in sinus surgery, reduction in mean daily OCS dose, adherence	Reduction in OCS pharmacy claims, Reduction in OCS bursts, reductions in NP-related and asthma-related healthcare costs. Asthma-related costs: total medical costs	Exacerbations: 70% NP-related reduction, 55% asthma-related reduction. OCS: 77% mean daily dose reduction, 43% pharmacy claims reduction, 59% burst reduction. Surgery: 43% sinus surgery reduction. Adherence: 70% achieved MPR/PDC ≥ 80%. Costs (asthma-related): 69% reduction at variable follow-up ($2,492 to $770 medical; $2,586 to $798 total) and 61–62% at 12-month follow-up ($961 medical; $995 total).	Payer/healthcare utilization perspective	12 months post-initiation (short-term) and variable follow-up ≥ 12 months (long-term).
Bhutani M (2017)	Reduction in exacerbations, exacerbation-free rate, improved ACQ and AQLQ scores	Reduced healthcare utilization: ED visits, hospitalizations, unscheduled visits	71% reduction in exacerbations, 56% remained exacerbation-free, improved ACQ and AQLQ score. Significantly reduced OCS use (2301.5 mg to 1130.0 mg annually), improved quality of life, The mean number of Emergency Department visits (0.2 ± 0.1 vs. 1.2 ± 0.1,), hospitalizations (0.1 ± 0.1 vs. 0.4 ± 0.1) and unscheduled healthcare professional visits (0.8 ± 0.2 vs. 3.1 ± 0.4) were all significantly reduced during the treatment follow up period. In addition, hospitalized subjects had a significantly reduced length of stay (0.6 ± 0.3 vs. 1.8 ± 0.3 days).	Healthcare system	12 months post-initation
Carpagnano et al. (2021)	Reduction in exacerbations; reduction in OCS daily dose, improvement pulmonary function	Treatment and drug cost difference omalizumab vs. mepolizumab	Switching to mepolizumab provided comprehensive clinical benefits with minimal economic impact.82.1% reduction in exacerbations; 77% reduction in OCS daily dose; 88% reduction in OCS-dependent patients; improved pulmonary function. Minimal cost difference: €12,239 (omalizumab) vs. €12,639 (mepolizumab). Drug cost increase (+€1,581) offset by savings in other areas (-€1,181) Switch justified in omalizumab non-responders	Healthcare system	12 months pre- and post-switch
Casale et al. (2019)	Exacerbation rate improvement, ACT score improvement	Hospitalizations reduction, reduction healthcare resource utilization	Exacerbation rate improvement (3.00 ± 3.28 to 0.78 ± 1.37), ACT score improvement (4.4 ± 4.9), 87% responder rate. Hospitalizations reduced by 81.9% (22.1% to 4.0%), reduced healthcare resource utilization	Health system and economic perspective	12 months pre-post treatment initiation
Casale et al. (2021)	Reduction in exacerbation, rate of hospitalization, OCS dose reduction	Reduced exacerbation-related HCRU: inpatient admissions, ED visits, outpatient visits, pharmacy claims	Mepolizumab provided clinical benefits across all 7 comorbidity subgroups studied. Exacerbations reduced 38–55%, hospitalizations reduced 57–83%, OCS dose reduction 29–38%. Reduced exacerbation-related HCRU: inpatient admissions, ED visits, outpatient visits, pharmacy claims all reduced > 50%	Health system and economic perspective	12 months pre-post treatment initiation
Chapman et al. (2024)	Reduction in exacerbations, reduction in OCS use, adherence	Reduction in asthma-related GP costs and visits, reduction in specialist costs and visits, reduction in ED visits, reduction in total HCRU	46.1% reduction in asthma exacerbations, 40.2% reduction in GP visits, 27.2% reduction in specialist visits, 52.1% reduction in ED visits, 33.1% reduction in OCS use. Significant reductions 37.1% in asthma-related GP costs, 36.8% reduction in specialist costs, 33.9% reduction in total HCRU costs excluding drugs (CAD$6,214 vs. CAD$4,109), high adherence (93.1% received ≥ 9 injections)	Healthcare system	12 months pre- and post-switch
Chen et al. (2016)	Reduction in asthma medication, exacerbations (31.2% to 11.8%), ER visits (1.13 ± 2.04 to 0.29 ± 0.83), hospitalizations	Cost reduction: ER expenses	60% patients improved with 8-point CSS cutoff; significant reductions in exacerbations, ER visits, hospitalizations. Reduction in asthma medication, exacerbations (31.2% to 11.8%), ER visits (1.13 ± 2.04 to 0.29 ± 0.83), hospitalizations. Cost reduction: ER expenses from 3934 to 2860 New Taiwan Dollar (NTD)	Healthcare system	12-month follow-up
Chen et al. (2023)	Reduction in exacerbations, reduction in OCS use	Asthma-related ED visits and hospitalizations	Biologic initiation associated with greater improvements than continuing HOCS alone in severe asthma patients. Biologics: 72.9% reduction in exacerbations (0.64 vs. 2.06), 2.2x more likely to achieve OCS <5 mg daily.Lower risk of asthma-related ED visits (RR 0.35) and hospitalizations (RR 0.31)	Healthcare system	20-month follow-up
Chung et al. (2022)	Reduction in exacerbations, reduction in OCS use	Reduction in HCRU, exacerbation-related costs decrease	55% reduction in exacerbation rates (3.25 to 1.47 per person-year), 41% had no exacerbations post-treatment. OCS-dependent patients decreased from 82% to 50%, 42–57% reduction in HCRU, exacerbation-related costs decreased by $6439 ($13,559 vs. $7120	Health system and economic perspective	12 months pre-post measurement
Entrenas Costa et al. (2019)	Exacerbation rate improvement, ACT score improvement, FEV1 improvement	ICER per avoided exacerbation (direct costs), and including indirect costs. Healthcare resources costs reduction	Omalizumab effective add-on therapy reducing key drivers of asthma costs including exacerbations, ED visits, and hospitalizations with favorable cost-effectiveness. Exacerbations reduced from 7.6 ± 7.4 to 1.4 ± 2.9, ACT score improved from 11.5 ± 3.4 to 19.9 ± 3.7, FEV1 improved + 10.8 points. ICER €1712 per avoided exacerbation (direct costs), €1607 including indirect costs. Healthcare resources costs reduced by €1235 per patient	Healthcare system	follow-up variabile
Gouder et al. (2015)	Reduction in exacerbations, reduction in OCS use, FEV1 improvement	Hospitalization costs reduction, unscheduled visits costs, sick days costs	Exacerbation reduction: -36.8%, ACT improvement: from 14 to 20 Steroid courses reduction: -62%, FEV1: non-significant improvement. Hospitalization costs: reduction from € 14,403 to €4,418, unscheduled visit costs decrease from € 3,890 to € 1,250, sick days costs registred 80% reduction	Healthcare system	53 weeks (1 year)
Humbert M et al. (2022)	Treatment persistence, discontinuation rates, asthma control, exacerbations	Reduction in HCRU, exacerbation-related costs decrease	Median treatment persistence 51.2 months (adults) and 53.7 months (children). 75% reduction in hospitalization rates, 30% reduction in OCS use at 2 years. Among controlled patients at discontinuation: 70%, 39%, 24% remained controlled at 1, 2, 3 years	Healthcare system	24 months
Jahnz-Różyk et al. (2018)	Reduction in exacerbations, exacerbation-free rate, improved ACQ and AQLQ scores, reduction in OCS use	Reduction in HCRU, exacerbation-related costs decrease and ED visits	Significant clinical improvements with omalizumab: 66% median reduction in exacerbation rate, 7.7 mg average OCS dose reduction, AQLQ score improvement + 1.86 points, ACQ score improvement + 1.45 points. 67% of mOCS patients achieved ≥ 50% OCS reduction, 53% eliminated mOCS. Mean treatment cost increased by 15,979 EUR/patient/year (baseline: 802 EUR/year to treatment: 16,781 EUR/year). Cost to avoid one exacerbation: 17,721 EUR. Other healthcare costs decreased due to fewer hospitalizations and ED visits	Healthcare system	12 months
Jackson et al. (2022)	Reduction in exacerbations, exacerbation-free rate, improved ACQ and AQLQ scores, reduction in OCS use	Reduced emergency healthcare utilization: ED visits decrease, hospitalizations, total healthcare resource utilization	Benralizumab highly effective regardless of previous biologic use, F81% reduction in exacerbation rate (4.8 to 0.9), 48% of patients with baseline exacerbations became exacerbation-free. 67% of mOCS patients achieved ≥ 50% OCS reduction, 53% eliminated mOCS. ACQ-6 improved from 3.0 to 2.1, AQLQ improved from 3.5 to 4.5. Reduced emergency healthcare utilization: ED visits decreased from 1.1 to 0.3, hospitalizations from 0.7 to 0.2. Healthcare resource utilization decreased similarly in biologic-naive and biologic-experienced patients	Healthcare system	48 months
Mosnaim et al. (2020)	Asthma exacerbations, hospitalizations, emergency department visits, corticosteroid bursts, asthma control	Healthcare costs (total, asthma-related, exacerbation-related), healthcare resource utilization	HEDIS attainment associated with 37% lower likelihood of exacerbations (OR: 0.63), 13% lower total costs, 41% lower exacerbation costs. 75.2% patients attained ≥ 1 HEDIS measure	Health system and economic perspective	24 months
Menzies-Gow et al. (2023)	Asthma exacerbations, hospitalizations, emergency department visits, lung function, quality of life	Healthcare utilization: unscheduled visits, telephone calls, ambulance transports, ED visits, hospitalizations, ICU days	Tezepelumab reduced: hospital exacerbations by 79%, hospitalizations by 85%, all-cause ED visits by 28%, all-cause hospitalizations by 59%. Zero ICU days vs. 31 in placebo group	Health system and economic perspective	24 months
Niven et al. (2016)	Oral corticosteroid dose, asthma exacerbations, lung function (FEV1), quality of life (AQLQ, ACT, EQ-5D), weight	ED visit decrease, hospital admissions rate, outpatient visits (excluding those for omalizumab), and bed days per patient	82.4% response rate. Reduced OCS dose by 1.61 mg/day, hospital exacerbations by 0.97/patient, improved FEV1 by 4.5%, AQLQ by 1.38 points. 21/93 unemployed patients gained employment. Emergency department visits decreased from 1.12 ± 1.71 to 0.37 ± 0.91 per patient, hospital admissions from 1.24 ± 1.64 to 0.56 ± 1.33, outpatient visits (excluding those for omalizumab) from 4.60 ± 2.48 to 1.60 ± 2.07, and bed days from 6.61 ± 9.73 to 3.39 ± 8.49 per patient	Health system and economic perspective	12 months pre- intervention + 24 months post intervention
Padilla-Galo et al. (2023)	Exacerbations, asthma control (ACT score), lung function (FEV1), oral corticosteroid use, biomarkers	Healthcare resource use: hospitalizations, emergency department visits, clinical remission achievement	85.6% reduction in exacerbations, 81.4% achieved zero exacerbations, 70.5% reduction in OCS dose, 52.8% complete OCS withdrawal, 43.7% achieved clinical remission	Clinical outcomes and economic/resource use	12 months
Padilla-Galo et al. (2021)	Exacerbations, asthma control (ACT), lung function (FEV1), oral corticosteroid use	Total annual healthcare costs, incremental cost-effectiveness ratios	Total annual cost: €11,544 (baseline) vs. €14,043 (benralizumab). ICER: €602 per avoided exacerbation, €983.86 per 3-point ACT increase. 88% reduction in severe exacerbations, 100% response rate	Health system and economic perspective	12 months
Tabaza et al. (2023)	Prevalence of treatment-related problems, reduction in OCS use	Total cost burden	Urgent need for pharmaceutical care interventions to reduce TRPs and associated costs. High prevalence of treatment-related problems (mean 4.7 TRPs during hospitalization, 2.0 at discharge). Total cost burden USD 766,046.8 (cost savings USD 30,919.3 + cost avoidance USD 734,179.9)	Clinical outcomes and economic/resource use	48 months
Tungay et al. (2023)	Life years gained, Quality-adjusted life years, Improved asthma status and quality of life with omalizumab	Per-patient costs, ICER per LY gained, ICER per QALY gained, willingness-to-pay threshold	Omalizumab in combination with standard of care is cost-effective for severe asthma from Turkish public payer perspective.Life years (LY): 21.26 vs. 13.60 years (difference 7.67 LY); Quality-adjusted life years (QALY): 13.35 vs. 10.08 (difference 3.27 QALYs); Improved asthma status and quality of life with omalizumab. Per-patient costs: ₺425,329.81 (OML + SoC) vs. ₺23,607.08 (SoC) - difference ₺401,722.74; ICER: ₺52,427.04 per LY gained, ₺122,675.57 per QALY gained; Cost-effective below ₺156,948 willingness-to-pay threshold	Payer/healthcare utilization perspective	12 months
Wechsler ME et al. (2021)	Primary: treatment response (excellent, clinically meaningful, partial, non-response). Secondary: clinical asthma exacerbations, FEV1, ACT scores, oral corticosteroid use, blood eosinophil levels	Healthcare resource utilization (hospitalizations, ER visits, urgent care visits, unscheduled outpatient visits)	58.6% had excellent response, 16.3% clinically meaningful response. Significant reduction in CAEs (86.0% to 40.5%, *p* < 0.001), improved FEV1 (65.1% to 73.1%, *p* < 0.001), improved ACT scores (13.8 to 18.6, *p* < 0.001). >50% discontinued maintenance OCS. Significant reductions in all HRU categories	Clinical outcomes and economic/resource use	12 months
Wenzel S et al. (2016)	Primary: Change in FEV1 at week 12 in patients with ≥ 300 eosinophils/µL. Secondary: FEV1% change, severe asthma exacerbation rate, ACQ-5, AQLQ, FeNO	Healthcare resource utilization assessed through exacerbation-related events (hospitalizations, emergency visits, systemic corticosteroid use)	Dupilumab significantly improved FEV1 (0.26 L difference vs. placebo in 200 mg q2w, *p* = 0.0008; 0.21 L in 300 mg q2w, *p* = 0.0063), reduced severe exacerbations by 70-80.7% in high eosinophil group, improved ACQ-5 and AQLQ scores. Benefits observed regardless of baseline eosinophil count. Most common AEs: upper respiratory infections, injection-site reactions	Clinical outcomes and economic/resource use	4 months
Yoshikawa H et al. (2016)	Hospital-free days (HFD), hospitalization reduction	Medical costs comparison pre vs. post-omalizumab, ICER	Median hospitalization decreased significantly (5 times to 0 times). ICER: JPY 20,868 per additional HFD. Cost was JPY 20,868 per additional HFD	Health system and economic perspective	48 months

ROBINS-I assessment showed that 76% of observational studies had serious risk of bias, mainly due to confounding and selection bias, limiting causal inference. While intervention classification and outcome measurement were generally robust, selective reporting and missing data raised concerns, leading to GRADE downgrading to moderate/low certainty and highlighting the need for prospective, well-controlled comparative studies.

### Qualitative Synthesis of the Scientific Evidence

#### Clinical Outcomes

Asthma Exacerbations

Across all biologic therapies, substantial reductions in asthma exacerbations were consistently observed. Al-Lehebi et al. [26] documented exacerbation reductions with mepolizumab ranging from 46.1 to 81%, reporting a 76% reduction in clinically significant exacerbations (*p* < 0.001). Moreover, the treatment improved asthma control substantially, with patients achieving well-controlled status (ACT score ≥ 20) increasing from 15.0 to 64.5%.

Omalizumab studies revealed exacerbation reductions between 66 and 82.1%. Arrobas et al. [27] reported an 82.1% reduction in exacerbations (from 8.2 to 1.5 per patient-year) with a 54.1% reduction in Global Evaluation of Treatment Effectiveness (GETE) score. Chen et al. [28] observed exacerbation rates declining from 31.2% to 11.8% with concomitant reductions in emergency room visits (1.13 ± 2.04 to 0.29 ± 0.83). Bhutani et al. conducted the ASTERIX observational study across 25 sites in Canada with 99 patients, demonstrating a 71% reduction in asthma exacerbations, with 56% of patients remaining exacerbation-free when compared to the year prior to study entry [29]. Niven et al. reported an 82.4% response rate at 16 weeks with hospital exacerbations decreasing by 0.97 exacerbations per patient (*p* < 0.001) in the 12-month post-omalizumab period compared to pre-treatment [30]. 

Benralizumab demonstrated remarkable efficacy with an 81% reduction in exacerbation rates (from 4.8 to 0.9 per patient-year), and 48% of patients with baseline exacerbations became exacerbation-free [24]. Chung et al. [31] reported a 55% reduction in exacerbation rates (3.25 to 1.47 per person-year, *p* < 0.001), with 41% of patients experiencing no post-treatment exacerbations. Padilla-Galo et al. [32] documented an 85.6% reduction in exacerbations, with 81.4% achieving zero exacerbations.

Dupilumab exhibited sustained efficacy in reducing exacerbations by 70–80.7% in patients with elevated eosinophil counts [33]. Tezepelumab reduced hospital-based exacerbations by 79% and hospitalizations by 85% compared to placebo in the NAVIGATOR trial [34].

Oral Corticosteroid Use

Significant reductions in oral corticosteroid (OCS) dependence were documented across multiple studies. Bagnasco et al. [35] reported a 61% reduction in OCS-dependent patients (from 79.2% to 33%) with mepolizumab, alongside a 4.73 mg reduction in daily OCS dose. Bernstein et al. [36] observed a 77% reduction in mean daily OCS dose (*p* < 0.001) and a 43% reduction in OCS pharmacy claims (*p* < 0.001).

Bhutani et al. [29] demonstrated that the mean total annual OCS dose was reduced from 2301.5 mg (prednisone equivalents) in the year prior to omalizumab to 1130.0 mg (*p* < 0.0001). Niven et al. [30] reported that the mean daily dose of OCS decreased by 1.61 mg per patient per day (95% CI −2.41 to −0.80; *p* < 0.001) comparing pre-omalizumab and post-omalizumab periods, with 21 out of 162 patients with complete employment data gaining employment and 6 patients losing employment in the 12-month post-omalizumab period.

Carpagnano et al. [37] documented good results with switching from omalizumab to mepolizumab, achieving a 77% reduction in OCS daily dose and an 88% reduction in OCS-dependent patients. Benralizumab studies demonstrated that 67% of patients on maintenance OCS achieved ≥ 50% OCS dose reduction, with 53% achieving complete OCS elimination. Jackson et al. [24] found that OCS-dependent patients decreased from 82 to 50% (*p* < 0.001).

Lung Function and Asthma Control

Improvements in forced expiratory volume in one second (FEV1) were consistently reported. Al-Lehebi et al. [26] documented improvement in FEV1 from 62.8 to 73.0% predicted with mepolizumab. Wenzel et al. [33] demonstrated significant FEV1 improvements with dupilumab (0.26 L difference versus placebo in the 200 mg every-2-weeks group, *p* = 0.0008).

Asthma Control Test (ACT) scores improved substantially across interventions. Entrenas Costa et al. [38] reported score improvements from 11.5 ± 3.4 to 19.9 ± 3.7 (*p* < 0.001) with omalizumab. Wechsler et al. [39] documented ACT score improvements from 13.8 to 18.6 (*p* < 0.001) with reslizumab. Gouder et al. [40] observed improvements from 14 to 20 (*p* < 0.001) with omalizumab over 53 weeks.

Quality of life improvements, measured by the Asthma Quality of Life Questionnaire (AQLQ), were consistently demonstrated. Jahnz-Różyk et al. [41] reported AQLQ score improvements of + 1.86 points with omalizumab. Jackson et al. [24] documented AQLQ improvements from 3.5 to 4.5 with benralizumab.

Treatment Persistence and Adherence

Long-term treatment persistence data revealed sustained biologic therapy utilization. Humbert et al. [25] reported median treatment persistence of 51.2 months in adults and 53.7 months in children for omalizumab in a nationwide French cohort. Among patients with controlled asthma at discontinuation, 70, 39, and 24% remained controlled at 1, 2, and 3 years respectively, suggesting durable benefits even after therapy cessation.

Treatment adherence rates were consistently high across studies. Al-Lehebi et al. [26] documented that 94.6% of patients maintained ≥ 90% treatment adherence with mepolizumab. Bernstein et al. [36] reported that 70% of patients achieved medication possession ratio/proportion of days covered (MPR/PDC) ≥ 80%. Jackson et al. [24] observed that 84.5% of patients remained on benralizumab treatment at 48 weeks.

Treatment Response and Predictors

Response rates to biologic therapies were substantial across studies. Casale et al. [42] reported an 87% responder rate with omalizumab regardless of biomarker status. Jahnz-Różyk et al. [41] found that 78.1% of patients were responders (≥ 50% reduction in exacerbations or maintenance OCS), with 57.2% classified as superresponders (eliminated exacerbations and maintenance OCS).

Wechsler et al. [39] documented that 58.6% of patients achieved excellent response to reslizumab, with an additional 16.3% showing clinically meaningful response. Significant reductions in clinically apparent exacerbations (CAEs) occurred from 86.0 to 40.5% (*p* < 0.001), with improvements in FEV1 from 65.1 to 73.1% (*p* < 0.001).

Early response assessment demonstrated clinical utility in predicting long-term treatment success. Jackson et al. reported that early response at 4 weeks with benralizumab had a positive predictive value of 82.9% for sustained clinical benefits. This finding supports the potential for early treatment assessment to guide therapeutic decision-making and resource allocation.

Clinical remission achievement represented an important outcome for severe asthma management. Padilla-Galo et al. [32] reported that 43.7% of benralizumab-treated patients achieved clinical remission, defined as absence of exacerbations, normal lung function, and discontinuation of OCS. This finding suggests that biologics may fundamentally alter disease trajectory in select patients beyond symptomatic control.

#### Economic and Organizational Outcomes

Healthcare system perspective was the most common economic evaluation approach (*n* = 11, 40.74%), followed by health system and economic perspectives (*n* = 8), and combined healthcare system and payer perspectives (*n* = 2). Two studies adopted a societal perspective incorporating productivity losses and indirect costs.

Healthcare Resource Utilization

High reductions in healthcare resource utilization (HCRU) were observed across all biologic therapies. Hospitalizations decreased substantially: Al-Lehebi et al. [26] reported reductions from 22.5 to 6.9%, Casale et al. [42] documented an 81.9% reduction in hospitalizations (from 22.1 to 4.0%) with omalizumab, and Menzies-Gow et al. [34] observed an 85% reduction in hospitalizations with tezepelumab.

Emergency department (ED) visits declined significantly. Bernstein et al. [36] reported a 59% reduction in ED visits with mepolizumab, Chapman et al. [43] documented a 52.1% reduction in ED visits (*p* < 0.001), and Chen et al. observed a 72.9% reduction in biologics-treated patients. Jackson et al. reported ED visits decreasing from 1.1 to 0.3 events per patient.

Outpatient healthcare utilization showed variable patterns. Chapman et al. reported a 40.2% reduction in general practitioner visits (*p* < 0.001) and a 27.2% reduction in specialist visits (*p* < 0.001) with mepolizumab. However, Al-Lehebi et al. noted increased physician visits despite reduced hospitalizations and ED visits, likely reflecting proactive monitoring in biologic-treated patients.

Cost-Effectiveness and Economic Impact

Cost-effectiveness analyses revealed favorable incremental cost-effectiveness ratios (ICERs) for biologic therapies despite their high acquisition costs. Arrobas et al. [27] calculated an ICER of €2,244 per exacerbation avoided and €1,750 per unit decrease in GETE score for omalizumab from a societal perspective. Entrenas Costa et al. [38] reported an ICER of €1712 per avoided exacerbation using direct costs, decreasing to €1,607 when including indirect costs.

Tugay et al. demonstrated cost-effectiveness for omalizumab in the Turkish context with an ICER of ₺52,427.04 per life-year gained and ₺122,675.57 per quality-adjusted life-year (QALY) gained, both below the willingness-to-pay threshold of ₺156,948. The study reported substantial gains of 7.67 life-years and 3.27 QALYs with omalizumab plus standard of care versus standard of care alone.

Bagnasco et al. [35] documented total savings of €2469 per patient excluding mepolizumab costs, with 62% of savings derived from exacerbation reduction and 33% from productivity increases. These savings funded 22% of mepolizumab’s annual cost, demonstrating partial economic self-sustainability through patient improvement. Working days lost decreased dramatically from 15 to 4 days per patient-year (*p* < 0.0001), with the proportion of patients losing working days declining from 63.2 to 31.2% (OR 0.253, *p* < 0.0001).

In the study of Yoshikawa et al. [44] the median hospitalization decreased significantly from 5 times to 0 times over the 2-year period. Omalizumab led to an estimated increase of 40.8 HFD per responder patient per 2 years, with an incremental cost-effectiveness ratio of JPY 20,868 per additional hospital-free day, demonstrating that omalizumab can reduce hospitalization costs in children with severe asthma in the Japanese healthcare context.

Carpagnano et al. [37] reported minimal cost differences when switching from omalizumab to mepolizumab, with costs of €12,239 versus €12,639 respectively. Drug cost increases (+€1581) were substantially offset by savings in other healthcare areas (−€1181), demonstrating economic neutrality for therapeutic switching in non-responders.

Chapman et al. [43] observed a 37.1% reduction in asthma-related general practitioner costs (*p* < 0.001), a 36.8% reduction in specialist costs (*p* < 0.001), and a 33.9% reduction in total HCRU costs excluding drugs (CAD$6214 versus CAD$4109) with mepolizumab in the Canadian publicly funded healthcare system. High treatment adherence (93.1% received ≥ 9 injections) contributed to these favorable outcomes.

Chung et al. [31] documented that exacerbation-related costs decreased by $6439 ($13,559 versus $7120, *p* < 0.001) with benralizumab, alongside 42–57% reductions in overall HCRU. Padilla-Galo et al. [45] reported an ICER of €602 per avoided exacerbation and €983.86 per 3-point ACT increase for benralizumab, with total annual costs of €14,043 compared to €11,544 at baseline.

Mosnaim et al. [46] found that achieving HEDIS (Healthcare Effectiveness Data Information Set) quality targets in persistent asthma reduced total healthcare costs by 13% while significantly lowering resource utilization (hospitalizations 4.9 vs. 7.3%; ED visits 9.6 vs. 18.2%). Clinical outcomes improved markedly, with patients requiring fewer systemic corticosteroid bursts (43.8 vs. 51.6%) and showing a 30% reduction in exacerbation odds (OR 0.70, *P* < 0.001).These results indicate that structured monitoring and specialist involvement optimize disease control, directly reducing the economic burden through better adherence.

## Discussion

The evidence demonstrates that biologic therapies targeting key inflammatory pathways achieve substantial clinical benefits and favorable economic outcomes despite high acquisition costs. Exacerbation reductions of 46–86% were consistently observed across all biologic agents, translating into decreases in healthcare resource utilization, oral corticosteroid dependence, and improvement in patient-centered outcomes including lung function, asthma control, and quality of life.

Precision asthma medicine requires integrating biomarkers, phenotype, comorbidities, and treatment history rather than relying on single thresholds. Comorbidity patterns, particularly chronic rhinosinusitis with nasal polyps, should guide biologic selection, often favoring anti-IL-5 therapies for optimal personalized outcomes [47, 48]. 

The substantial OCS reduction and elimination rates (53–67% achieving ≥ 50% reduction) represent a critically important safety and quality-of-life benefit. Chronic OCS use associates with numerous adverse effects including osteoporosis, diabetes, hypertension, cataracts, and increased infection risk [49, 50]. This represents an important opportunity for future pharmacoeconomic analyses to quantify the indirect benefits of OCS reduction on comorbidity burden and long-term health outcomes [47].

A central finding of this review is also that, while biologic therapies substantially increase pharmaceutical expenditures, they generate significant offsets through reduced healthcare resource utilization. Hospitalization reductions of 57–85%, emergency department visit decreases of 52–72%, and substantial declines in unscheduled outpatient visits collectively diminish the economic burden of uncontrolled severe asthma. These findings align with health economic principles demonstrating that interventions targeting high-cost, high-utilization patient populations can achieve cost-effectiveness despite premium pricing [51]. 

Economic modeling studies show favorable cost-effectiveness for biologics when accounting for healthcare resource offsets. Spanish analyses [52] demonstrate benralizumab achieves the highest quality-adjusted life years (52.21 QALYs) at lowest cost (€56,094/patient) with superior exacerbation reductions (2.87 over 5 years). Omalizumab real-world data yields incremental cost-effectiveness ratios of €1,712 per exacerbation avoided (direct costs) or €1607 (including productivity losses). However, reported ICERs vary widely (€602–€122,676 per outcome unit) due to differences in healthcare pricing, comparators, and methodologies. In addition, willingness-to-pay thresholds differ across countries, which can affect the interpretation of cost-effectiveness. Standardization of health economic evaluation methods would improve cross-study comparability and resource allocation decisions. Productivity gains—particularly the 73% reduction in work absence days—represent substantial societal benefits frequently undervalued in analyses [53]. From a societal perspective incorporating indirect costs, biologics demonstrate stronger economic value, especially in working-age populations where productivity losses substantially impact total burden [54].

Another aspect emerge is about the high treatment persistence (median 51–54 months) and adherence rates (70–94.6% with ≥ 80–90% adherence) observed in real-world settings contrast with adherence challenges commonly encountered with inhaled therapies [55]. This difference likely reflects multiple factors including injectable administration in healthcare settings, intensive patient selection and education, and dramatic symptomatic improvements reinforcing adherence.

Access to biologics varies widely due to formulary restrictions, authorization requirements, and reimbursement policies across health systems. The reviewed evidence originates predominantly from high-income countries (USA, Western Europe, Canada), with limited data from middle-income settings and none from low-income countries, restricting understanding of cost-effectiveness and feasibility in resource-constrained environments where asthma burden remains substantial.

The biologics evaluated target distinct but overlapping inflammatory pathways: IgE (omalizumab), IL-5 pathway (mepolizumab, reslizumab, benralizumab), IL-4/IL-13 pathway (dupilumab), and thymic stromal lymphopoietin (tezepelumab). Matching patient inflammatory endotypes to targeted therapies represents the promise of precision medicine in asthma [56, 57]. However, the evidence base provides limited guidance on biomarker-driven treatment algorithms, particularly for patients without clear eosinophilic phenotype or with mixed inflammatory patterns [58].

Methodological Considerations, Strengths and Limitations

Self-controlled pre-post designs, which dominate the reviewed literature, present methodological limitations including regression to the mean, time trends, and absence of control groups, hampering causal inference. Ethical and practical objections restrict RCT use in severe asthma, resulting in only two such studies identified. While quasi-experimental methods like propensity score matching (e.g., GLITTER study) mitigate confounding, their use is rare, and residual bias persists. Heterogeneity in outcome definitions and economic measures limits cross-study comparability and meta-analysis. Potential publication bias exists, with a lack of negative or neutral studies reported and limited search of unpublished data. Follow-up was mainly limited to 12 months, studies with extended follow-up (24–48 months) provide more robust evidence on sustained effectiveness but remain minority observations. Long-term registries and observational cohorts are needed to address this evidence gap.

## Conclusions

Real-world evidence demonstrates that biologic therapies effectively translate trial efficacy into routine practice, reducing exacerbations, corticosteroid use, and costly acute events while improving patient outcomes. These data, capturing diverse populations and real-life resource utilization, offer the most policy-relevant foundation for optimizing severe asthma care beyond registrational trial constraints. From a health-system perspective, the economic burden of asthma—especially when severe or uncontrolled—should be continuously monitored using routine data sources (claims, electronic health records, registries) to quantify direct and indirect costs. Within this framework, biologics should be framed not merely as high-cost therapies, but as potentially cost-offsetting interventions when targeted to the appropriate phenotype and evaluated through early response assessments and harmonized outcome measures across centers.

Translating biologic benefits into routine care requires strengthening service organization through clear referral pathways to severe-asthma centers, standardized phenotype/biomarker assessment, integrated comorbidity management, and real-world dashboards monitoring outcomes, corticosteroid exposure, adherence, resource use, and total costs. Future research should strengthen the evidence base through head-to-head comparative effectiveness studies, biomarker-guided algorithms, long-term safety and effectiveness evaluations, pediatric data generation, and economic analyses incorporating societal perspectives, thereby supporting precision care, policy decisions, and more efficient resource allocation.

## Data Availability

The study materials are available from the corresponding author upon reasonable request.
